# Towards a taxonomy for integrated care: a mixed-methods study

**DOI:** 10.5334/ijic.1513

**Published:** 2015-03-04

**Authors:** Pim P. Valentijn, Inge C. Boesveld, Denise M. van der Klauw, Dirk Ruwaard, Jeroen N. Struijs, Johanna J.W. Molema, Marc A. Bruijnzeels, Hubertus JM. Vrijhoef

**Affiliations:** Scientific Centre for Care and Welfare (Tranzo), Tilburg University, Tilburg, The Netherlands; The Netherlands Expert Centre Integrated Primary Care, Jan van Es Institute, Almere, The Netherlands; The Netherlands Expert Centre Integrated Primary Care, Jan van Es Institute, Almere, The Netherlands; TNO, Leiden, The Netherlands; Public Health and Health Care Innovation, Department of Health Services Research, Faculty of Health, Medicine and Life Sciences, School for Public Health and Primary Care, Maastricht University, Maastricht, The Netherlands; Department of Quality of Care and Health Economics, Centre for Nutrition, Prevention and Health Services, National Institute for Public Health and the Environment, Bilthoven, The Netherlands; TNO, Leiden, The Netherlands; The Netherlands Expert Centre Integrated Primary Care, Jan van Es Institute, Almere, The Netherlands; Chronic Care, Scientific Centre for Care and Welfare (Tranzo), Tilburg University, Tilburg, The Netherlands; Health Systems and Policy, Saw Swee Hock School of Public Health, National University of Singapore, Singapore, Singapore

**Keywords:** integrated care, primary care, Delphi study, classification, literature review, taxonomy, coordinated care

## Abstract

**Introduction:**

Building integrated services in a primary care setting is considered an essential important strategy for establishing a high-quality and affordable health care system. The theoretical foundations of such integrated service models are described by the Rainbow Model of Integrated Care, which distinguishes six integration dimensions (clinical, professional, organisational, system, functional and normative integration). The aim of the present study is to refine the Rainbow Model of Integrated Care by developing a taxonomy that specifies the underlying key features of the six dimensions.

**Methods:**

First, a literature review was conducted to identify features for achieving integrated service delivery. Second, a thematic analysis method was used to develop a taxonomy of key features organised into the dimensions of the Rainbow Model of Integrated Care. Finally, the appropriateness of the key features was tested in a Delphi study among Dutch experts.

**Results:**

The taxonomy consists of 59 key features distributed across the six integration dimensions of the Rainbow Model of Integrated Care. Key features associated with the clinical, professional, organisational and normative dimensions were considered appropriate by the experts. Key features linked to the functional and system dimensions were considered less appropriate.

**Discussion:**

This study contributes to the ongoing debate of defining the concept and typology of integrated care. This taxonomy provides a development agenda for establishing an accepted scientific framework of integrated care from an end-user, professional, managerial and policy perspective.

## Introduction

Integrated care is increasingly being promoted as a means for improving accessibility, affordability and the quality of health care, especially for people with complex needs [1,2]. Essential for achieving desired health outcomes and limiting costs, primary care is considered the cornerstone of such integrated care approaches [3–5]. However, despite the increasing popularity of developing integrated service models in a primary care setting a solid knowledge base is lacking [6]. In particular, the knowledge base is hampered by the lack of common terminology and typology regarding integrated care [2].

In a recent article, we proposed the Rainbow Model of Integrated Care [7] as a framework to unravel the complexity of integrated care. The Rainbow Model of Integrated Care distinguishes four dimensions that play inter-connected roles on the macro- (system integration), meso- (organisational, professional) and micro-level (clinical integration) and two more dimensions (functional and normative integration) that enable the connectivity between the various integration levels (see Table 1). The Rainbow Model of Integrated Care is considered useful for understanding the complex and multidimensional nature of integrated care [8]. However, the underlying key features of these six integrated care dimensions are yet unknown. Insight into the underlying key features is essential for achieving a common operational understanding of integrated care and for contributing to programme implementation, policy formulation and research analysis.

Consequently, there is a need for a common taxonomy that can classify the broad spectrum of integrated care approaches. A taxonomy is a formal system to classify a multifaceted complex phenomena [9], and, in this study, this complex phenomena is ‘integrated care’. A taxonomy applied to integrated care would facilitate the description and comparison of different integrated care programmes which is essential for translating research findings and evidence into practical tools for policy and practical implementation. Likewise, this taxonomy is needed to support effective deployment of integrated service models in a primary care setting. The aim of the present study is to contribute to a better understanding and operational consensus regarding the concept of integrated care by addressing the following objectives:

- Based on a literature review, define the Rainbow Model of Integrated Care by developing a taxonomy that specifies the underlying key features of the six integrated care dimensions;
- Investigate the appropriateness of the key features to achieve integrated care in a primary care setting among a group of experts from The Netherlands.

## Theory and methods

### Theoretical background

Integrated care, as defined by Leutz (1999), is a broad inter-sectorial system approach that aims to align the health care system (acute, primary medical and skilled) with other human service systems (e.g. long-term care, education and vocational and housing services) [10]. Primary care, as stated in the Alma-Ata declaration of 1978 [11], describes a similar inter-sectorial system approach with a distinct community and socio-political focus. However, theoretical discourses on integrated care and primary care as a broad inter-sectorial system approach have failed to produce practical relevance for practices and policies [12]. To bridge this gap, a common taxonomy is needed to move towards a clearer operational consensus regarding integrated care as a whole.

In this article, *integrated care* refers to ambulatory care settings in which a network of multiple professionals and organisations across the health and social care system provide accessible, comprehensive and coordinated services to a population in a community. Based on the Rainbow Model of Integrated Care, integration of services can be achieved at a system (system integration), institutional (organisational integration), professional (professional integration) and service (clinical integration) levels. The distinctions between these different levels provide comprehensive insight into the features needed to achieve integrated care within a system. Throughout this paper, we refer to *features of integrated care* as entities, processes or structures which operate in particular contexts to achieve integrated care.

## Methods

We applied a mixed-method approach consisting of: (1) a literature review, (2) a thematic analysis to develop a taxonomy, and (3) a Delphi study to test the relevance of the taxonomy among a group of experts from The Netherlands. Because no patients were involved in this study, ethical approval was not required under Dutch law.

### Literature review

A literature review was conducted to identify the key features that could be used to organise integrated care. The databases Cochrane Library, Medline, Scopus and Business Source Premier were searched for articles published during the period from January 2002 to December 2012 and written in English. Because the present study specifically focused on the organisation of integrated care, the focus of the literature review was narrowed to system (inter-sectorial), organisational (inter-organisational) and professional (inter-professional) models of integration. The following search terms were used: ‘delivery of health care’, ‘integrated service system’, ‘integrated systems’, ‘inter-organisational collaboration’, ‘inter-organisational cooperation’, ‘inter-professional collaboration’ or ‘inter-professional work’ and ‘quality model’. The detailed search and selection strategy appears in ‘Additional File 1’.

To be included, publications had to meet the following criteria: (1) a description of a theory or model of inter-sectorial, inter-organisational or inter-professional service delivery, (2) a description of the features (underlying entities, processes or structures) used to achieve integrated service delivery. Publications were excluded that reported clinical interventions and a main focus on clinical outcome measures (e.g. HbA1c levels or hospital re-admission rates) or process indicators (e.g. percentage of patients receiving treatment).

Two researchers (PV and IB) independently reviewed the titles and abstracts. Only when both of the researchers independently found the title and abstracts relevant, the article was retrieved. Any disagreements between the researchers were resolved by consensus. For every included publication, we briefly described the theory or model, the study design and the main research theme of the article.

### Thematic analysis

A three-step thematic analysis method was used [13,14] to synthesise the results of the literature review and to develop a taxonomy of key features. First, two researchers (PV and IB) generated an initial list of features from the included articles. To be initially included, features had to meet the following three criteria: (1) Relevance (related to achieving clinical, professional, organisational, system, functional and/or normative integration); (2) Theoretical foundation (presence of a theory, model or logic was described in the article); and (3) Clarity (clear definition or descriptions of the reported features). Thereafter, the initial list of features was categorised across the six dimensions of the Rainbow Model of Integrated Care according to the description of each feature as reported in the literature. Any disagreements between the researchers were resolved by consensus. Second, three researchers (PV, IB and MB) independently assessed the compiled taxonomy and combined features into overarching key features within each integrated care dimension. During three discussion rounds, overarching key features were compared for agreement among the researchers and iterative revisions were made. Also, features that were identical or nearly identical were merged and descriptions were formed during these rounds. Finally, two external researchers (DK and JM) and a research assistant independently reviewed the preliminary taxonomy and offered feedback for refining the descriptions of the key features. Feedback included suggestions for merging and/or reorganising specific key features within and between the different dimensions. PV and IB summarised the feedback and revised the taxonomy accordingly.

### Delphi study

A Delphi study was conducted using the RAND UCLA appropriateness method [15]. In the first round, a self-administered questionnaire was used, and in the second round the experts revalued their first round score after a group discussion in a physical meeting. The aim of the second discussion round was to determine if ratings were different due to real disagreement or due to a misunderstanding or misinterpretation of the features [15]. A purposive sampling strategy was used to identify experts with experience in practice or science regarding the deployment of integrated service models in a primary care setting. The following selection criteria were used for the experts: a scientific (doing research) or practical (working in a professional or service organisation) background regarding the organisation of integrated primary care delivery. Based on this criteria, experts were selected to ensure that a balanced number of both were represented. We decided not to include stakeholders like patients and health insurers in order to minimalize conflict of interest in the procedure (e.g. strategic behaviour of the experts because they are dependent on these stakeholders). Thirty-three experts were approached by e-mail and/or telephone and invited to participate. We then included experts that indicated that they would be available to participate in both consensus rounds. Following the RAND UCLA appropriateness method, between 9 and 15 experts were ultimately selected [15].

During round one, the experts received written information on the research aims and details of the Delphi procedure. After they committed to participate, they received a link to an online questionnaire and were asked to rate the appropriateness of each feature for achieving integrated care in a primary care setting on a 9-point Likert-scale, ranging from 1 (completely irrelevant) to 9 (extremely relevant). The features were randomly presented to the experts to avoid order and information bias, which could potentially transpire especially if the features were presented in the order of the six Rainbow Model of Integrated Care dimensions. In addition, all experts were invited to suggest possible rephrasing of the descriptions of the features and add new features. After one week, reminders were sent by e-mail to non-responders.

In round two, a face-to-face meeting of the expert panel took place which was chaired by one of the researchers (MB) with experience in facilitating group discussions. The meeting's goal was to discuss the results of round one and revalidate the features. Based on the results of round one, a summary report was provided to the experts with the following key feedback information: (1) respondents’ own ratings in round one, (2) median agreement rating, (3) summary of qualitative comments, as well as (4) whether consensus was achieved at round one. Because of time, we decided to only discuss the features that did not reach agreement in the first round. We clustered these features by theme (e.g. leadership, strategy, value creation, external environment) and asked the highest and lowest scoring panel member to clarify his or her consideration. Next, a short discussion among all group members took place. Finally, the experts were asked to, once again, individually rate the features that were not agreed upon in the first round.

### Data Analysis

The data extracted during the thematic analysis process were listed and analysed using MS Excel. The criteria of the RAND UCLA appropriateness method were used to analyse the data from the Delphi study [15]. We categorised the overall panel median as follows: 1–3 as inappropriate, 4–6 as equivocal and 7–9 as appropriate. Agreement signified that ≥70% of panellists’ ratings were within the same 3-point region (that is, 1–3, 4–6 or 7–9) as the observed median. A feature was defined as ‘appropriate’ with an overall panel median score of ≥7 and a level of agreement of ≥70% within the 3-point region 7–9. A panel median of 4–6 or median with a consensus of ≤70% within the same 3-point region was defined as ‘equivocal’. A feature with a panel median of 1–3 and a level of agreement of ≥70% within the 3-point region 1–3 was defined as ‘inappropriate’. The decision rules used in both rounds are shown in Table 2. Values were computed using SPSS version 21 for Windows (IBM Statistics).

## Results

### Literature review

**Figure 1.**
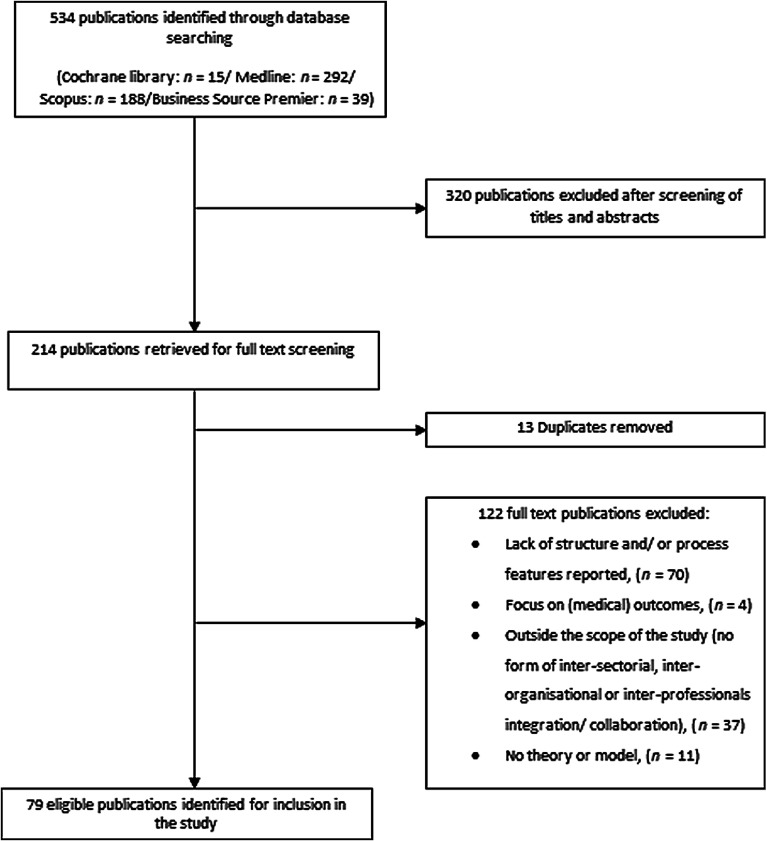
Flowchart of the literature search

Our literature search yielded 534 potentially relevant publications (Figure 1). After screening titles and abstracts, we retrieved 214 potentially relevant publications for their full-text. We excluded 320 publications because they were not considered relevant to the current study. Out of the 214 eligible publications, 13 duplicates were removed and another 122 publications were excluded for reasons given in Figure 1. Finally, a total of 79 publications were included in the literature review.

Most of the included publications were based on empirical studies (66%, *n* = 52); other publications were based on non-empirical study designs (27%, *n* = 27). Table 3 lists the main research topics of the included publications. Approximately one-third of the publications focused on inter-organisational collaboration (30%, *n* = 24); other common themes were integrated service delivery (18%, *n* = 14), inter-professional collaboration (11%, *n* = 9) and inter-organisational learning (10%, *n* = 8). More descriptive information can be found in ‘Additional File 2’.

### Thematic analysis

**Figure 2.**
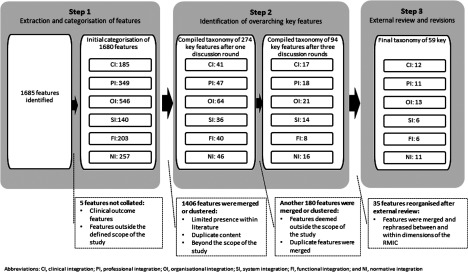
Flowchart of the thematic analysis process

Figure 2 provides a schematic overview of the thematic analyse process employed to synthesise the literature and to develop the taxonomy of key features. The reasons for removing features at each step of the thematic analysis process appear in the dashed boxes in Figure 2. First, an initial list of 1685 features was extracted from the 79 included publications of which 1680 features were categorised across the six dimensions of the Rainbow Model of Integrated Care (see Step 1 in Figure 2). Second, the compiled taxonomy of 1680 features was reviewed by three authors (PV, MB and IB) to identify the broader and overarching key features per dimension. During the first discussion round, 274 key features were identified by the three reviewers. There was little disagreement among the three authors on combining features to form over-reaching key features, and any existing disagreement was easily resolved by discussion. During these subsequent discussion phases, most features were merged within each dimension due to similar or nearly identical content. After the third discussion round, 94 potential key features were identified (see Step 2 in Figure 2). Finally, the compiled taxonomy was reviewed by two external reviewers (DK and JM) and a research assistant. Based on the feedback of the reviewers, the features were further merged and refined within and between the six dimensions based on their similar content (see Step 3 in Figure 2). The resulting taxonomy of 59 key features is shown in Table 4.

### Delphi study

In total, 14 persons participated in the first round of the expert panel (response rate 40%). The main reason experts choose not to participate was their inability to be available for the second face-to-face meeting. The panel was a balanced group of experts with a scientific (50%, *n* = 7) or practical (50%, *n* = 7) background. The panellists had a mean age of 45.4 years (SD: 11.3, range: 28–68) and a mean of 11.6 years (SD: 8.8, range 4–40) of experience in integrated care initiatives. Based on round 1, 25 of the 59 key features were considered appropriate (overall panel median of 7–9 and consensus of ≥70% within the same 3-point region, see Table 5). Thirty-four features were rated as equivocal for achieving integrated care in a primary care setting (overall panel median of 4–6 or median with consensus of ≤70% within the same 3-point region). None of the key features were considered inappropriate (overall panel median of 1–3 and consensus of ≥70% within the same 3-point region), and the experts did not propose any new features.

In the second round, one expert with practical experience and three scientific experts could not attend, resulting in a 10-member panel. This had no major impact on the composition of the panel compared to round 1. The panellists in round 2 had a mean age of 47.5 years (SD: 11.5, range: 28–68) and a mean of 10.9 years (SD: 8.8, range 4–40) of experience. Discussion during the second round on the 34 equivocal features resulted in an extra nine features rated as appropriate. Within the clinical dimension, the key features *interaction between professional and client* (no. 6) and *population needs* (no. 11) and within the organisational dimension the key features *interest management* (no. 27) and *managerial leadership* (no. 32) were rated appropriate after the second round. Within functional dimension the key feature *regular feedback of performance indicators* (no. 48) reached consensus after the second round. Furthermore, within the normative dimension, the key features *sense of urgency* (no. 50), *visionary leadership* (no. 53), *quality features of the informal collaboration* (no. 55) and *linking cultures* (no. 56) were rated appropriate. Twenty-four key features remained equivocal after the second round, and only one key feature was rated as inappropriate, namely *reputation* (no. 57) within the normative dimension.

The results in Table 5 show that the appropriate key features are unevenly distributed across the six dimensions of the taxonomy. In particular, within the dimension of system integration, *stakeholder management* (no. 40) was the only key feature considered appropriate. Additionally, within the dimension of functional integration, half of the key features that refer to key support functions were considered equivocal by the experts; *human resource management* (no. 43), *resource management* (no. 45) and *support systems and services* (no. 46). Particularly noteworthy within the dimension of clinical integration is that five of its key features (nos. 3, 4, 8, 10 and 12) were considered equivocal by the experts for achieving integrated care in a primary care setting.

Corresponding features across the dimensions of the taxonomy, such as value creation and leadership, also showed an uneven pattern. For example, key features concerning value creation (nos. 21, 24 and 37) were only considered appropriate from a ‘professional’ integration perspective (no. 21) and not from an organisational or system integration perspective. Moreover, key features regarding leadership (nos. 19, 32 and 53) were only considered appropriate from an organisational perspective and normative integration perspective, but not from a professional integration perspective (no. 19).

## Discussion

This study aimed to define a taxonomy to contribute to the ongoing debate of specifying the concept of integrated care using a theory-driven mixed-method approach. Based on the theoretical foundations of the Rainbow Model of Integrated Care [7] and a literature review, we developed a taxonomy of 59 key features distributed across six integration dimensions (clinical, professional, organisational, system, functional and normative integration). A Delphi study further indicated that 34 of these 59 key features were considered appropriate for achieving integrated care in a primary care setting. The majority of the key features associated with the clinical, professionals, organisational and normative dimensions of integration were considered appropriate for achieving integration in a primary care setting. Key features associated with the functional and system dimensions of integration were considered less appropriate.

The results of the Delphi study indicated that the key features associated with the professional and organisational dimensions were considered appropriate for achieving integration in a primary care context. This result is not surprising as the professional and organisational perspective regarding integrated care has been the prime focus of practice, science and policies [2,95]. Moreover, the experts considered the key features associated with the normative dimension of the taxonomy as appropriate enablers for achieving integrated service models in a primary care setting. While existing integrated care theories, models and instruments tend to have a limited focus on these ‘soft enabling features’ of integrated care [96–99], it is, nevertheless, very likely that these normative or soft features play a crucial role in the development of various complex inter-sectorial, inter-organisational and inter-professional service models of integration. Although the existing academic literature also suggests that functional integration (e.g. information management systems) are important enabling mechanisms for achieving integrated care [100], fewer of these key features were considered appropriate when compared to the normative key features.

An intriguing finding was that, despite socio-political influences being frequently mentioned as essential preconditions for achieving integrated care [2,5,101,102], the experts considered most of the key features associated with the system integration perspective as equivocal for achieving integration in a primary care setting. A possible explanation for this inconsistency might be found in the composition of our expert panel, as we did not explicitly include experts with a macro-policy background (e.g. policymakers or health insurers). This might have resulted in the underexposure of the macro-system perspective in the results of our Delphi study. On the other hand, at the micro-clinical level, the experts considered the key features related to the involvement of clients and patients as equivocal for achieving integration in a primary care setting. Most of the experts considered integrated service delivery as a ‘backstage’ process for the benefit of clients and patients. This opinion does not concur with the current academic literature that highlights the key position of patients in the integration process [2,103–106]. This inconsistency might be explained by the fact that patients and clients were not included in the expert panel. The lack of interest being placed at the macro- (system) and micro- (patient) levels made us aware that integrated care can be defined from multiple perspectives depending on the actors involved (e.g. patients, professionals, managers and policymakers) [2]. This indicates the need to develop assessment tools which take into account these various perspectives (e.g. a 360-degree feedback method) when evaluating the performance of an integrate care approach.

### Strengths and weaknesses

The strength of this study is its theory-driven mixed-method approach. The taxonomy is theoretically grounded on the Rainbow Model of Integrated Care [7] and has a solid base in the current academic literature. The strength of the thematic analysis procedure lies in its potential to synthesise and identify common features across a heterogeneous mix of publications [13,14,107]. The Delphi study added substantially towards consensus-based terminology regarding the development of integrated service models within a primary care context.

A limitation of the study relates to the composition of our expert panel, as patients and experts with a macro-policy background were not included. As noted earlier, the lack of emphasis on key features associated with the macro- (system) perspective and patient involvement in achieving integrated care might be due to the composition of our expert panel. We are aware of the fact that this form of selection bias might be present in our Delphi study. However, it appears difficult to include all perspectives in one expert panel without introducing other serious forms of bias (e.g. conflict of interests) [15,108]. We did not explicitly included experts with a macro-policy background because their presence could influence the (strategic) behaviour of the practice experts, as they are (financially) dependent on these experts for the continuity of their practices. Besides, the results of the Delphi study also confirm that an expert opinion regarding integrated care has a more limited scope compared to a broad theoretical discourse of integrated care [2,109–111]. Another limitation of this study relates to the subjective interpretation process during the thematic analyses method. Although the synthesis process was systematic and independently verifiable, subjective judgements of the researchers could have had an impact on the construction of the key features of the taxonomy [14,112].

Another challenge in the present study relates to the complex nature of integrated care, which can never be fully rationalised or standardised [113–115]. However, the vast majority of research on integrated care is based on an industrial-quality improvement logic which holds that quality standardisation leads to better outcomes and allows for more systematic evaluations [115]. Researchers (ourselves included) often struggle with the delicate balance of collating, analysing and synthesising findings which are academically defensible against research methods that do not necessarily appreciate the underlying epistemological assumptions of integrated care. We have attempted to use a more pragmatic approach to address this gap. By developing a taxonomy that holds much promise, our study aimed to potentially guide the modelling and development of pioneering research approaches across traditional disciplinary boundaries in order to reveal the complex inter-relationships at a system, institutional, professional and service level [115]. We think further debate about the underlying epistemological assumptions and methodology and quality considerations of integrated care would be extremely useful. We invite other scholars to explore with us the philosophical basis of integrated care and to establish an agreed upon ‘state of the science’.

### Implications for practice and research

Our study fills an important gap in the knowledge base of the concept of integrated care. The key features of the taxonomy provide a crucial differentiation to describe and analyse various types of integrated service models (ranging from comprehensive towards more selective). In this way, the taxonomy might be a valuable contribution for health care professionals, managers, patient organisations, health care service purchasers and policymakers involved in the complex organisation of integrated service delivery. The taxonomy can also serve as set of hypotheses for future empirical investigation. Moreover, our study is a vital step towards the creation of a common language and an understanding of the concept of integrated care. Future research should explore the relevance and acceptability of our taxonomy in order to establish a common terminology regarding integrated care. In addition, researchers could examine the categorisation of the key features among the dimensions of integrated care in order to further refine the current taxonomy.

## Conclusion

This study established a taxonomy for integrated care based on the theoretical foundations of the Rainbow Model of Integrated Care. The taxonomy can be considered a first step towards a common typology and operational consensus regarding integrated care. More work is needed to develop research methodologies that take into account the various integration processes from an end-user, professional, managerial and policy perspective in a synergetic way. For this purpose, the taxonomy has established a further developmental agenda for both research and practice.

## Figures and Tables

**Table 1.**
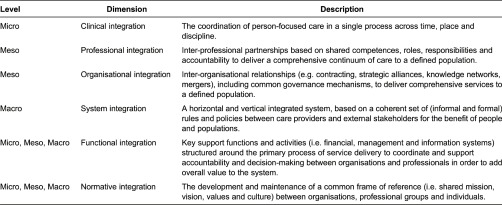
Integrated care dimensions of the Rainbow Model of Integrated Care

Adopted from Valentijn et al. [7].

**Table 2.**
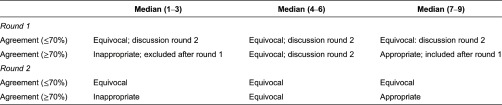
Decision rules of the Delphi study

**Table 3.**
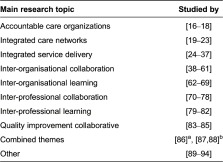
Research themes of the included publications

^a^Combination of the research themes inter-professional and inter-organisational collaboration.

^b^Combination of the research themes inter-organisational and inter-professional learning.

**Table 4.**
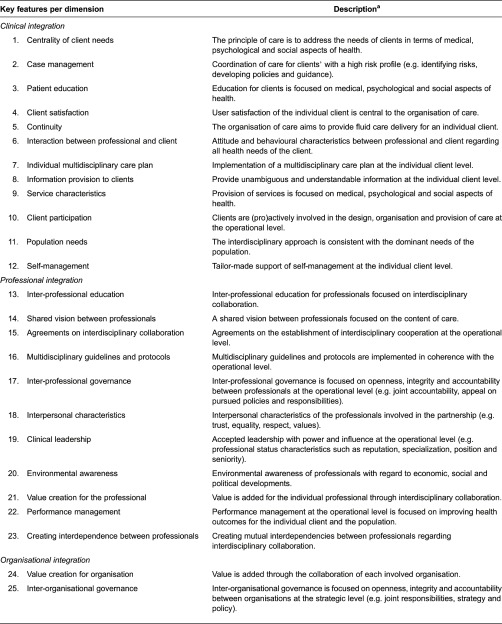

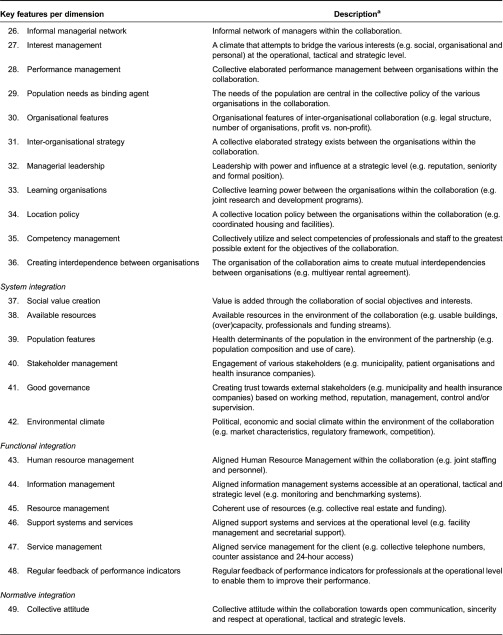

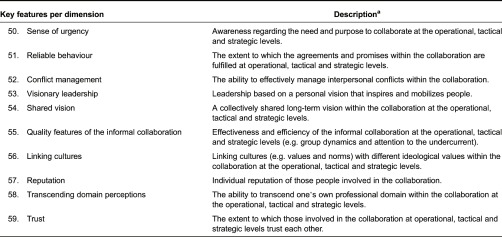
Taxonomy of 59 key features

^a^Descriptions are derived from the literature, and were refined during Step 3 of the thematic analysis process.

**Table 5.**
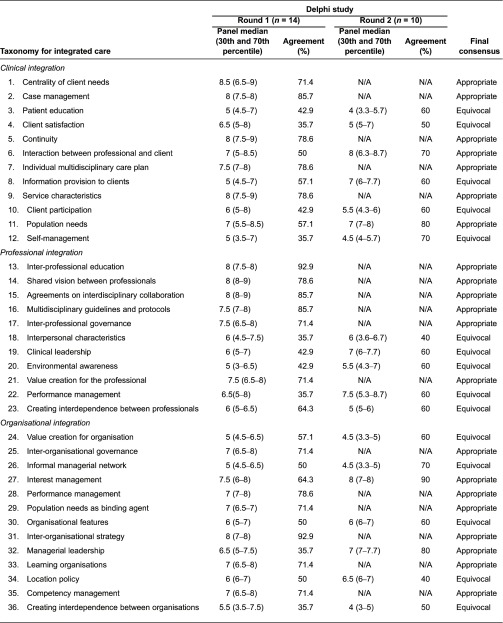

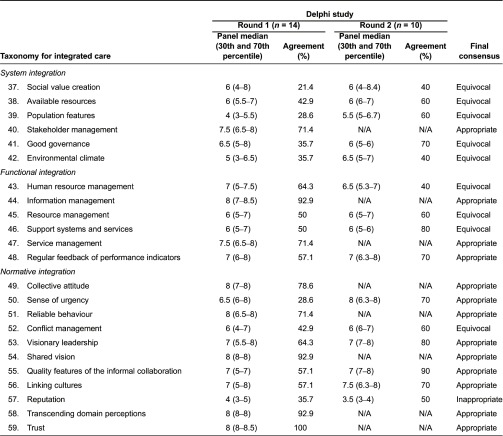
Results of the Delphi study

N/A, not applicable as a consensus had already been reached.
